# Cerebellar Transcranial Direct Current Stimulation Modulates Corticospinal Excitability During Motor Training

**DOI:** 10.3389/fnhum.2018.00118

**Published:** 2018-04-09

**Authors:** Rebekah L. S. Summers, Mo Chen, Andrea Hatch, Teresa J. Kimberley

**Affiliations:** ^1^Divisions of Physical Therapy and Rehabilitation Science, Department of Rehabilitation Medicine, School of Medicine, University of Minnesota, Minneapolis, MN, United States; ^2^Department of Psychiatry, University of Minnesota, Minneapolis, MN, United States; ^3^Non-invasive Neuromodulation Laboratory, MnDRIVE Initiative, University of Minnesota, Minneapolis, MN, United States

**Keywords:** cerebellum, motor performance, transcranial direct current stimulation, corticospinal excitability, transcranial magnetic stimulation, motor evoked potential

## Abstract

**Background**: Cerebellar activity can be modulated using cerebellar transcranial direct current stimulation (ctDCS) and, when applied concurrently with task training, has been shown to facilitate cognitive and motor performance. However, how ctDCS facilitates motor performance is not fully understood.

**Objective/Hypothesis**: To assess the electrophysiological and motor performance effects of ctDCS applied during motor training.

**Methods**: Fourteen healthy adults (age 28.8 ± 10.5 years) were randomly assigned to complete one session of finger tracking training with either simultaneous bilateral anodal or sham ctDCS. Training was completed in two 15 min epochs with a 5-min break (total 30 min stimulation, 2 mA). Tracking accuracy and corticospinal and intracortical excitability were measured immediately before and after the training period. Motor cortical excitability measures included resting motor threshold (RMT), motor evoked potential (MEP) amplitude, cortical silent period (CSP) and short interval intracortical inhibition (SICI).

**Results**: There was a significant interaction of Group * Time for MEP amplitude and CSP duration (*p* < 0.01). *Post hoc* analysis revealed MEP amplitude was increased in the sham group (*p* < 0.01), indicating increased corticospinal excitability from baseline while the anodal group displayed a decrease in MEP amplitude (*p* = 0.023) and prolongation of CSP duration (*p* < 0.01). SICI and RMT remained unchanged following ctDCS and training. Task accuracy was improved in both groups at post-test with a significant effect of Time (*p* < 0.01); however, there was no effect of Group (*p* = 0.45) or interaction of Group * Time (*p* = 0.83). During training, there was a significant effect of Block (*p* < 0.01) but no significant effect of Group or interaction effect (*p* > 0.06).

**Conclusions**: ctDCS applied during task training is capable of modulating or interfering with practice-related changes in corticospinal excitability without disrupting performance improvement.

## Introduction

The cerebellum is involved in a multitude of motor-cognitive tasks such as spatial attention, sequence learning, mental rotation and the control of the body in movement tasks (Molinari and Leggio, 2007). Recent literature investigating non-invasive forms of brain stimulation, such as transcranial direct current stimulation (tDCS), demonstrates that cerebellar transcranial direct current stimulation (ctDCS) can enhance cognitive and motor performance in healthy adults (Hardwick and Celnik, 2014; Cantarero et al., 2015; Oldrati and Schutter, 2017). tDCS is a method of non-invasive brain stimulation that modulates spontaneous cellular activity via weak electrical currents applied to the scalp (Bindman et al., 1964; Nitsche et al., 2003). In general, cathodal tDCS reduces spontaneous cellular activity while anodal stimulation increases cellular activity (Nitsche and Paulus, 2000). Cerebellar stimulation may be a promising therapeutic intervention for people with movement disorders or as an adjunct to rehabilitative therapies to enhance motor learning (Ferrucci et al., 2016). Specifically, ctDCS may help to regulate cerebellar excitability, which could be impaired in disorders such as tremor, dystonia and ataxia. However, published reports of ctDCS effectiveness are inconsistent and the validity of ctDCS for clinical use has been debated (Jalali et al., 2017). In healthy adults, anodal ctDCS applied during task training results in enhanced learning and motor performance (Oldrati and Schutter, 2017). Yet, it is unknown how ctDCS facilitates motor performance and learning.

Motor training generates practice-related, neuroplastic changes in the motor cortex with reports of increased motor evoked potential (MEP) amplitude (Pascual-Leone et al., 1994; Perez et al., 2004) and reduction of short interval intracortical inhibition (SICI) and cerebellar-brain inhibition (CBI; Perez et al., 2004; Jayaram et al., 2011; Schlerf et al., 2012; Baarbé et al., 2014; Spampinato and Celnik, 2017). During task learning, cerebellar-brain-inhibition has been shown to decrease proportionally to the magnitude of learning (Jayaram et al., 2011; Spampinato and Celnik, 2017). Depression of CBI suggests a potential reduction from cerebellar output, Purkinje cells. A reduction in Purkinje cell activity would theoretically disinhibit deep cerebellar nuclei and promote increased excitability along thalamic-cortical pathways. Similarly, when tDCS is applied to the cerebellum without motor activity, cerebellar-brain-inhibition has been shown to decrease following both anodal and cathodal ctDCS, rTMS and tACS (Tremblay et al., 2016). However, no change in resting motor threshold (RMT) or MEP amplitude have been reported following ctDCS alone (for review see Tremblay et al., 2016).

Although changes in brain excitability have been explored following ctDCS alone, changes in corticospinal excitability following concurrent ctDCS and task training has been minimally evaluated and electrophysiological measures of motor cortex excitability are rarely assessed. Visual-spatial motor learning and performance is a task requiring cerebellar involvement (Miall et al., 2001; Miall and Reckess, 2002) and has been shown to be facilitated with ctDCS (Sriraman et al., 2014; Cantarero et al., 2015). When corticospinal excitability is assessed following simultaneous ctDCS and motor training, both anodal and cathodal ctDCS have been reported to facilitate MEP amplitudes (Shah et al., 2013), while others have found no change in corticospinal excitability (Sriraman et al., 2014; Craig and Doumas, 2017). Based on these three studies, it is unclear what or if any electrophysiological changes are influenced by ctDCS when combined with motor activity, despite the potential of ctDCS to enhance behavior (Grimaldi et al., 2014; Celnik, 2015; Oldrati and Schutter, 2017). Interestingly, changes in electrophysiology from tDCS are state dependent (Thirugnanasambandam et al., 2011; Antal et al., 2014), meaning task execution during ctDCS may reverse or modify expected changes of electrophysiological outcomes.

To determine how ctDCS influences facilitated behaviors, it is essential to define how motor cortex excitability is regulated following combined motor training and ctDCS. We chose to evaluate intracortical and corticospinal excitability as these are outcomes that have shown significant changes with motor training. The purpose of this investigation was to: (1) assess the feasibility of applying stimulation with concurrent motor training at durations similar to that of a session of therapeutic rehabilitative training; and (2) assess the electrophysiological and motor performance effects of ctDCS applied during motor training. We hypothesized that ctDCS would be feasible and that anodal ctDCS concurrent with motor training tasks would enhance motor performance compared to sham ctDCS.

## Materials and Methods

### Participants

Fourteen healthy adults ages 18–52 (Mean ± SD: 28.8 ± 10.5, 8 male) participated in the study. Hand dominance was determined using the Edinburgh Handedness Inventory (Oldfield, 1971). Exclusion criteria included medications acting on gamma-aminobutyric acid and dopaminergic neurotransmission, implanted devices, history of seizure in the last 2 years, pregnancy, or any neurologic or psychiatric conditions. This study was carried out in accordance with the recommendations of the University of Minnesota Institutional Review Board Institutional Review Board and Clinical Translational Science Institute with written informed consent from all subjects. All subjects gave written informed consent in accordance with the Declaration of Helsinki. The protocol was approved by the University of Minnesota Institutional Review Board.

### Experimental Design

A blinded, randomized, pre-test/post-test study design was used. Participants were randomly assigned to two study groups: anodal or sham ctDCS applied during task training. Pre-test and post-test assessments consisted of tracking accuracy index (AI) on a visual-spatial motor task and transcranial magnetic stimulation (TMS) corticospinal excitability measures. Participants and testers were blinded to group allocation. An unblinded investigator not involved in testing pre-programmed the device for group allocation.

### Electromyography and Transcranial Magnetic Stimulation

Corticospinal excitability was assessed using a 70-mm figure-of-eight TMS coil connected to a Magstim 200^2^ stimulator (The Magstim Company Ltd., Carmarthenshire, UK). Electromyography (EMG) responses were recorded from the dominant first dorsal interosseous (FDI) with a pair of stainless steel disc electrodes (101085, Natus neurology Inc., Pleasanton, CA, USA). The active electrode was positioned on the muscle belly of the FDI and the reference electrode was placed on the first metacarpophalangeal joint. EMG signals were amplified by bipolar EMG amplifiers (Y03–2, Motion Lab Systems, Inc., Baton Rouge, LA, USA) with a gain of ×300 and band-pass filter (20–2000 Hz), then digitized by an analog-to-digital convertor (NI 9234, National Instruments, Austin, TX, USA) with a 24-bit resolution at a sampling rate of 6.4 kHz. The ground electrode (TD-431, Discount Disposables, St. Albans, VT, USA) was wrapped around the dominant hand’s wrist similar to previously established methods (Summers et al., 2017).

During testing, participants were seated in a semi-reclined chair. The TMS coil was positioned over the primary motor cortex (M1) with the handle directed posterolaterally 45° to the mid-sagittal line of the head. The optimal cortical site to elicit an MEP in the FDI was determined using single pulse magnetic stimuli and the RMT was established at this site. The RMT threshold was defined as the lowest intensity needed to elicit an MEP amplitude greater than 50 μV five out of ten trials (Rossini et al., 2015), while the 1 mV threshold was defined similarly with an MEP of 1 mV in 5 out of 10 trials. RMT and 1 mV threshold was re-measured at post-test to account for potential practice-related changes in excitability. The location of TMS was tracked using stereotactic neuronavigation guided stimulation (BrainSight, Rogue Research Inc., Montreal, QC, Canada).

Corticospinal excitability measures included: short-interval intracortical inhibition (SICI), cortical silent period (CSP), and MEP amplitude, and were collected according to previously established methods (Chen et al., 2015). To measure SICI, a subthreshold (80% RMT) conditioning stimulus was given, followed by a 3 ms interstimulus interval and a subsequent suprathreshold (1 mV threshold) testing stimulus. Ten trials of conditioned SICI responses and ten unconditioned single pulse responses at 1 mV threshold were recorded for a total of 20 randomized trials. For CSP testing, the participant’s maximum voluntary contraction was recorded over three trials by abducting the right index finger against a solid surface. During CSP testing, participants maintained an isometric contraction of the FDI at 20% of the maximum voluntary isometric contraction EMG intensity, using visual feedback from a custom LabView program. During the contraction, a single pulse was delivered to the FDI cortical location previously identified as the motor hotspot. Ten trials of CSP measurements were collected. To measure MEP amplitude, 10 trials were recorded using 120% of the pre-test RMT.

### Visual Spatial Motor Task

Motor training was implemented using an index finger extension and flexion tracking training program (Carey et al., 1994). The tracking program was displayed before the participant on a laptop computer (Dell Computer Corporation, Round Rock, TX, USA). Participants were oriented to the motor task with one trial of passive finger movement to familiarize the participant to the task, followed by two active trials of tracking sine wave patterns. Finger movement was tracked using a potentiometer (Waters Manufacturing and Company, Wayland, MA, USA) aligned to each participant’s metacarpophalangeal joint of the dominant index finger. The voltage signal from the finger tracking device was directed to the laptop using an analog-to-digital converter (Interactive Structures, Inc., Bala-Cynwyd, PA, USA) with a sampling rate of 60 Hz.

Each pre-test and post-test assessment of tracking accuracy consisted of 10 trials on a random type waveform, varying in frequency, amplitude and velocity of the cursor. To challenge aspects of spatial orientation in the motor task we included stimulus-response compatible and incompatible trials. Of the 10 trials, five were in a stimulus-response compatible form and five were in an incompatible form. Compatible condition: forearm pronated, finger movement in the sagittal plane; incompatible condition: forearm semi-pronated or neutral, finger movement in the horizontal plane.

Tracking training with simultaneous application of ctDCS used novel waveforms not used for pre and post accuracy testing, and were varied in amplitude, frequency and cursor speed. Training was implemented in six 4-min blocks (24 trials of 10 s) with 1 min rest between blocks to promote learning and avoid negative motivational factors. Training was performed for 15 min (3 blocks), followed by a 5-min rest break to avoid fatigue, after which another 15-min epoch (3 blocks) was completed. Feedback of performance was given to the participant by the software program displaying an AI score after every five trials for knowledge of performance and to serve as a motivational tool.

### Transcranial Direct Current Stimulation

**Figure 1:**
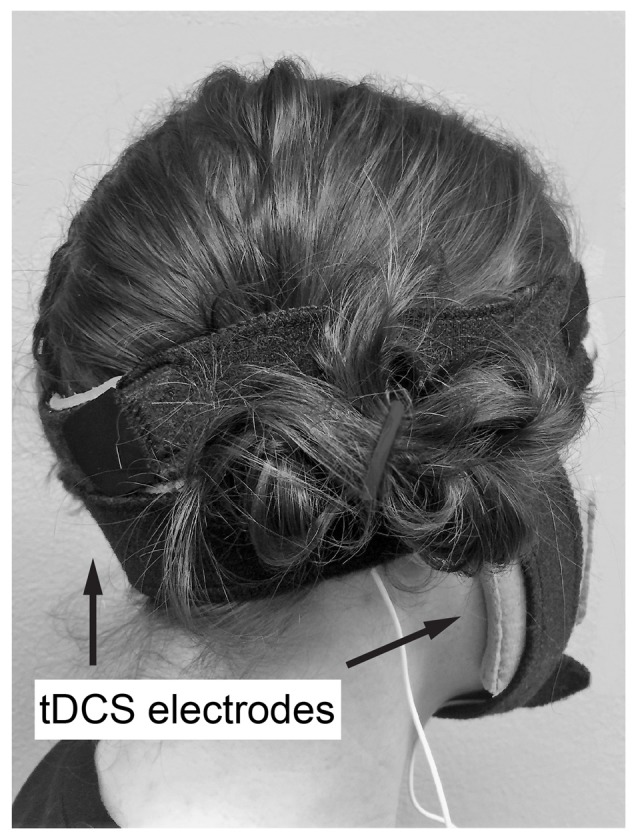
Example of transcranial direct current stimulation (tDCS) electrode placement. Active electrode at a midpoint between the level of the mastoid process and inion, along the posterior midline of the head, reference on buccinator muscle ipsilateral to the trained hand.

Anodal or sham ctDCS was delivered to the bilateral cerebellar hemispheres by centering the 70 × 100 mm active electrode at a midpoint between the level of the mastoid process and inion, along the posterior midline of the head (Figure 1). Active electrode placement was intended to target the vermal region and bilateral hemispheres of the cerebellum (Ferrucci et al., 2013). The reference electrode (50 × 70 mm) was placed on the buccinator muscle ipsilateral to the training hand. Stimulation was applied with a constant current of 2 mA using a direct current stimulator (TCT Research Limited, Hong Kong) in two 15-min epochs during training with a 5 min break between epochs. Following standard practice for sham tDCS, sham tDCS was applied using an automated setting on the tDCS unit, which ramps down current intensity to 0 after 30 s (Gandiga et al., 2006). At the end of the study session, participant blinding was assessed by asking participants which form of stimulation they believed they received (real or sham).

### Data Processing

Accuracy of the tracking task was calculated by using the following calculation for all trials (testing and training trials) with the AI: 100(P − E)/P, where E is the root-mean-square error between the target line and the performance line and P is the size of the target pattern, defined as the root-mean-square difference between the wave and the midline separating the wave (Carey, 1990).

For the corticospinal excitability measures, peak-to-peak amplitude of each MEP for single and paired pulse responses was calculated. Each SICI response was divided by the mean value of the ten single pulses for pre-test and post-test respectively. For the CSP, the EMG data were rectified and a 10-ms moving SD calculation was applied to the data with the onset of the CSP being the time point of the stimulus delivery. The average of the moving SD of the pre-stimulus data (−100 ms to −5 ms) was used as a threshold to determine the offset of the CSP, which was defined as the point that the moving SD value returned to the pre-stimulus level. To calculate statistical power, AI normalized change scores for all participants were calculated using a linear transformation as: *Normalized change score* = $(Y-{\bar{x}}_{pre})/{\bar{x}}_{pre}$ where, *Y* is the individual values of the post-test and ${\bar{x}}_{pre}$ is the mean value of the individual values of the pre-test.

### Data Analysis

Normality was evaluated using Shapiro-Wilks test on data within each group for comparisons. Pre-test data was compared between groups with independent t-tests in normally distributed samples and Independent-samples Mann-Whitney U tests for non-normal data. A mixed model analysis of variance (ANOVA) with Subject as the random factor, Group (anodal/sham) and Time (pre/post) as fixed factors, was used to assess changes within and between groups. *Post hoc* tests were completed using Tukey HSD on pairwise comparisons as appropriate.

Accuracy improvement during training trials was assessed using a mixed ANOVA with between subject factor: group, and within subject factor: training block. Mauchly’s test of sphericity and Levene’s test for homogeneity of variances was used prior to ANOVA. Blinding effectiveness was assessed by reporting the number of participants who reported the correct group allocation (real or sham). Similarly, adverse events were assessed in each participant. Significance level for all statistical tests was *p* < 0.05.

## Results

All participants completed the study (13 right hand dominant; anodal group: *n* = 7, mean age ± SD: 27.1 ± 10.5, 3 males; sham: *n* = 7, age 28.9 ± 10.5, 5 males). The left-handed individual was randomized to the sham group. Three participants were excluded in the MEP amplitude analysis due to data loss (participant 1, 3, 4). Two participants were excluded from the tracking AI analysis due to data loss (participant 1) or due to self-reported inability to attend to the task for the required duration (participant 13). When assessing participant blinding, participants reported the correct group assignment (real or sham tDCS) in 4/7 in the real group and 1/7 in the sham group. Expected non-significant adverse events included metallic taste in the mouth and itching sensations near the electrode (1/7 in the active group and 1/7 in the sham group). There were no differences between groups on any excitability or behavioral measure at pre-test (*p* = 0.553–0.099). Group pre-test and post-test data are reported in Table 1.

**Table 1:** Pre-test and Post-test data.

Outcome	Sham			Anodal		
	Pre	Post	*N*	Pre	Post	*N*
MEP Amplitude	861.70 (361.8)	1702.30 (1090)	6	1009.80 (432.9)	708.60 (392.7)	5
RMT	41.60 (4.4)	43.10 (6.0)	7	44.70 (8.7)	46.10 (8.8)	7
SICI	0.20 (0.17)	0.39 (0.23)	7	0.27 (0.29)	0.29 (0.26)	7
CSP	149.30 (23.0)	155.9 (30.0)	7	143.20 (12.0)	166.70 (39.5)	7
Tracking AI	11.40 (9.8)	27.1 (7.6)	6	6.40 (11.5)	25.90 (9.4)	6

*Data are mean (standard deviation). MEP, motor evoked potential, RMT, resting motor threshold; SICI, short-interval intracortical inhibition; CSP, cortical silent period; AI, Accuracy Index; N, number of participants included*.

### Tracking Accuracy Results

**Figure 2:**
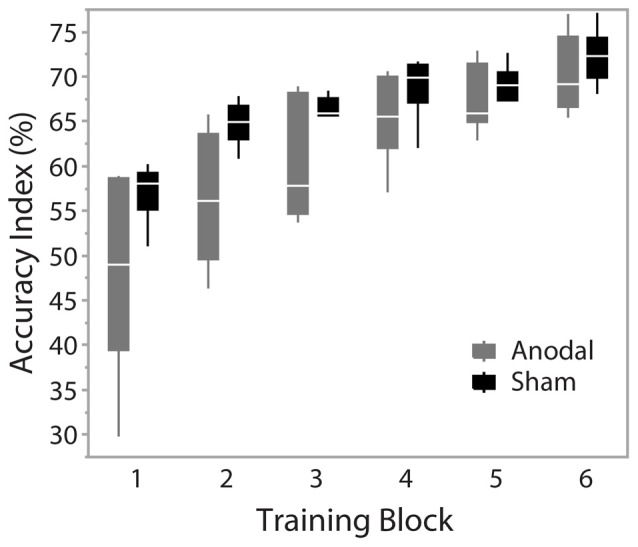
Accuracy across training blocks. Blocks 1–6 represent training blocks with simultaneous cerebellar tDCS. A 5-min rest period was given between block 3 and 4. Data are median and range with 95% confidence intervals.

**Figure 3:**
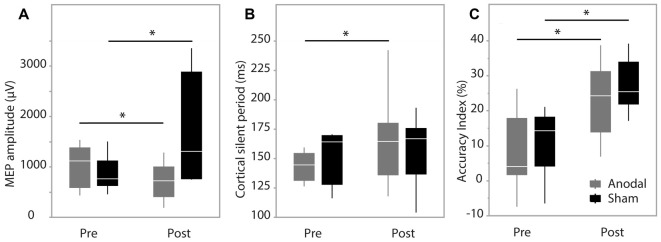
Pre-test and post-test data. **(A)** Motor evoked potential (MEP) amplitude; **(B)** cortical silent period (CSP) duration; **(C)** accuracy index (AI). Data are median and range with 95% confidence intervals. *Significant result, *p* < 0.05.

During training trials, there was a significant effect of training block on accuracy scores (*F*_(1.74,17.386)_ = 48.403, *p* < 0.001), indicating improved performance with training duration. Group was not a significant factor (*F*_(1,10)_ = 4.217, *p* = 0.067). There was no interaction effect between training block and group (*F*_(1.74,17.386)_ = 2.754, *p* = 0.097), suggesting no behavioral advantage of applying anodal ctDCS during task training (Figure 2). Tracking accuracy was significantly increased within both groups at post-test with a significant effect of time (*F*_(1,245)_ = 93.83, *p* < 0.0001) suggesting that all participants’ accuracy improved with training (Figure 3C). There was no group effect (*F*_(1,245)_ = 0.62, *p* = 0.45) or interaction of group*time (*F*_(1,245)_ = 0.047, *p* = 0.83).

### Corticospinal Excitability Results

MEP amplitude analysis produced no significant effect of group (*F*_(1,9)_ = 1.62, *p* = 0.24), while there was a significant effect of time (*F*_(1,202.2)_ = 10.48, *p* = 0.0014) and interaction between group*time (*F*_(1,202.2)_ = 43.93, *p* < 0.0001). *Post hoc* analysis revealed MEP amplitude was increased in the sham group at post-test (*p* < 0.0001) and decreased in the anodal group at post-test (*p* = 0.0228; Figure 3A). Group was not a significant factor when assessing changes in CSP (*F*_(1,12)_ = 0.028, *p* = 0.87), while there was a significant effect of time (*F*_(1,263)_ = 31.49, *p* < 0.0001) and a significant interaction between group*time (*F*_(1,263)_ = 9.66, *p* = 0.0021). Pairwise comparisons were significant only in the anodal group (*p* < 0.0001) indicating a prolongation of CSP duration in the anodal group (Figure 3B). SICI was not modulated during the study with no effect of group (*F*_(1,12)_ = 0.017, *p* = 0.89), time (*F*_(1,264)_ = 3.36, *p* = 0.068) or interaction effect (*F*_(1,264)_ = 1.98, *p* = 0.16).

## Discussion

The primary finding of this work is that ctDCS applied during finger tracking training is capable of modulating or interfering with practice-related changes in corticospinal excitability without disrupting performance improvement. Motor training has been shown to lead to practice-related changes in the motor cortex, including a facilitated MEP amplitude response (Pascual-Leone et al., 1994; Perez et al., 2004; Spampinato and Celnik, 2017). Our work replicated these reports as demonstrated by an increased MEP amplitude following motor training in the sham stimulation group. In contrast, anodal ctDCS group demonstrated decreased excitability, suggesting that the ctDCS interfered with expected practice-related excitability increases. Our results also suggest an effect of ctDCS to increase CSP duration. Importantly, these changes were observed despite stability in RMT and SICI. To the best of our knowledge, this is the first comprehensive assessment of intracortical and corticospinal excitability following combined ctDCS and motor training.

Changes in motor cortex excitability have rarely been assessed following ctDCS combined with training, thus comparison to prior reports is limited. Ankle tracking combined with either anodal or cathodal ctDCS facilitated the MEP amplitude in the tibialis anterior muscle (Shah et al., 2013), which is in disagreement to our results. However, in that work, the increase in excitability reported may reflect expected learning excitability changes in the motor cortex, unaffected by ctDCS, as relatively low current intensity and duration were applied (1 mA for 15 min). In support of this consideration, it has been suggested that a minimum of 2 mA may be needed to effectively modulate cerebellar targets with tDCS (van Dun et al., 2016). Two other studies of anodal ctDCS (1–2 mA) applied during motor training have produced no change in corticospinal excitability (Sriraman et al., 2014; Craig and Doumas, 2017). All three studies reporting TMS outcomes following ctDCS combined with training have only assessed training periods up to 15–20 min. We speculate the 30-min training session applied in this study may have allowed the influences of ctDCS on motor cortex excitability to manifest more fully. One study in agreement with our work assessed motor imagery-related changes in motor cortex excitability before and after ctDCS. Motor imagery requires activation of the motor cortex and facilitates MEP amplitudes. Following anodal ctDCS, motor imagery had reduced influence on MEP amplitudes compared to sham ctDCS (Cengiz and Boran, 2016). These results suggest that not only is the cerebellum receptive to tDCS neuromodulation, but that it also has an inhibitory influence on motor cortex excitability that is evident when the motor cortex is simultaneously activated.

Our finding of reduced corticospinal excitability when training was combined with anodal ctDCS is intriguing, and we offer two, non-exclusive hypotheses as explanation: (1) enhanced inhibitory tone from Purkinje cells due to anodal ctDCS; or (2) state dependent plasticity whereby ctDCS combined with motor practice allows cerebellar-thalamic-cortical pathways to be modulated. In support of the first hypothesis, enhanced Purkinje cell firing has been suggested as a primary effect of ctDCS (Ferrucci et al., 2016), leading to increased inhibitory output from Purkinje cells, downregulation of output from deep cerebellar nuclei, and disfacilitation along dentato-thalamo-cortical pathways. In support of the second hypothesis, tDCS is highly dependent on the physiologic state of the neuronal region stimulated (Thirugnanasambandam et al., 2011; Antal et al., 2014). ctDCS alone has been shown to induce changes in cerebellar brain inhibition (Galea et al., 2009; Jayaram et al., 2011); yet, no significant effects on the M1 threshold or MEP amplitude have been reported when assessing offline effects of ctDCS (Tremblay et al., 2016). Online effects have been demonstrated, whereby anodal ctDCS reduced MEP amplitudes, but only when tested during a low level muscle contraction using an anterior to posterior TMS current (Hamada et al., 2014). Here, we provide evidence that concurrent task training with anodal ctDCS lead to offline changes in motor cortex excitability that outlast the training period. Concurrent motor training may place cerebellar-M1 pathways in a state that is receptive to weak electrical currents, thereby modifying the state of the neuronal pools targeted by tDCS. We are unable to further dissect these hypotheses as those tests were not the focus of the experiment.

### Absence of Learning Benefit

No learning advantage from tDCS was found during the training period or at post-test in this study. This finding contradicts prior reports of improved performance from anodal ctDCS applied during task training (Oldrati and Schutter, 2017). An explanation for lack of effect may be due the duration of training, selected task, or electrode placement. A plateau in scores was present in blocks 6 and 7, which may indicate a max training effect or decreased effort during the training period. The task may not have probed specific aspects of motor control or the location of stimulation may not be optimal for the cerebellar region regulating performance of the specific task. Cerebellar mediated aspects of visual-motor training may include feed-forward prediction (Miall and Jenkinson, 2005). Protocols manipulating predictive timing from visual and motor output may be one method to tease out specific aspects of motor learning that may be modulated with non-invasive cerebellar stimulation. In contrast to our work, some studies assessing combined tracking training with ctDCS applied ctDCS unilaterally (Shah et al., 2013; Sriraman et al., 2014), while we used a bilateral or vermal cerebellar montage with the active electrode along the midline of the head (Ferrucci et al., 2013). We chose to target the midline regions of the cerebellum because vermal regions are involved in visual-motor training (Miall et al., 2001; Miall and Jenkinson, 2005).

### Limitations

Conclusions are limited by a small sample size and lack of a cerebellar specific outcome measure. Thus, results may be used to inform future work evaluating offline effects of ctDCS during motor training and require replication in a larger sample. A power analysis was performed on AI normalized change scores using the data reported here, with alpha = 0.05 and power = 0.8, Hedges *d* = 0.389, suggesting a small effect size (Portney and Watkins, 2009) of anodal ctDCS on task accuracy. This effect size is comparable to other studies evaluating anodal ctDCS to enhance behavioral outcomes (for review see Oldrati and Schutter, 2017). It is noteworthy that our sample did reveal significant changes in corticospinal excitability despite no differences in tracking accuracy, indicating an ability of ctDCS to influence neurophysiologic substrates of motor training. No outcome measure was used to assess cerebellar function directly, however; cerebellar modulation can be inferred from the significant influence of active ctDCS on motor cortex excitability compared to sham ctDCS. Future work should seek to utilize a combination of appropriate measures of cerebellar function, electrophysiological outcomes (e.g., electroencephalography, corticospinal excitability, brainstem response curve), and behavioral outcomes (e.g., error rate, task learning, feed-forward prediction) to facilitate the understanding of ctDCS and potential clinical applications.

## Conclusion

ctDCS combined with task training is a safe and feasible paradigm to pair with task training. This work suggests that motor training with simultaneous ctDCS results in modulation of motor cortex excitability compared to motor training with sham ctDCS. The results fail to demonstrate an association of performance and changes in electrophysiology. The electrophysiological effects of ctDCS are state dependent and rarely assessed, thus; there is a continued need for mechanistic investigations and comprehensive neurophysiologic assessments.

## Author Contributions

RLSS, MC and TJK contributed to the conception and design of the study. RLSS organized the database and wrote the first draft of the manuscript. RLSS and MC performed the statistical analysis. RLSS, MC, TJK and AH contributed to manuscript revision, read and approved the submitted version.

## Conflict of Interest Statement

The authors declare that the research was conducted in the absence of any commercial or financial relationships that could be construed as a potential conflict of interest.
